# A new core–shell-type nanoparticle loaded with paclitaxel/norcantharidin and modified with APRPG enhances anti-tumor effects in hepatocellular carcinoma

**DOI:** 10.3389/fonc.2022.932156

**Published:** 2022-09-14

**Authors:** Ming-Hua Xie, Zai-Lin Fu, Ai-Lian Hua, Ji-Fang Zhou, Qian Chen, Jian-Bo Li, Shen Yao, Xin-Jun Cai, Min Ge, Li Zhou, Jia Wu

**Affiliations:** ^1^Department of Pharmacy, First People’s Hospital of Linping District, Hangzhou, China; ^2^Department of Pharmacy, Zhejiang Integrated Traditional Chinese and Western Medicine Hospital, Hangzhou, China; ^3^Department of Oncology, First People’s Hospital of Linping District, Hangzhou, China

**Keywords:** hepatocellular carcinoma, paclitaxel, norcantharidin, core–shell lipid nanoparticles, APRPG

## Abstract

Nanoparticle delivery systems have been shown to improve the therapeutic efficacy of anti-cancer drugs, including a variety of drugs for the treatment of hepatocellular carcinoma (HCC). However, the current systems show some limitations, and the delivery of more effective nanoparticle systems for anti-HCC drugs with better targeting ability are needed. Here, we created paclitaxel (PTX)/norcantharidin (NCTD)-loaded core–shell lipid nanoparticles modified with a tumor neovasculature-targeted peptide (Ala-Pro-Arg-Pro-Gly, APRPG) and investigated their anti-tumor effects in HCC. Core–shell-type lipid nanoparticles (PTX/NCTD-APRPG-NPs) were established by combining poly(lactic-co-glycolic acid) (PLGA)-wrapped PTX with phospholipid-wrapped NCTD, followed by modification with APRPG. For comparison, PTX-loaded PLGA nanoparticles (PTX-NPs) and PTX/NCTD-loaded core–shell-type nanoparticles without APRPG (PTX/NCTD-NPs) were prepared. The *in vitro* and *in vivo* anti-tumor effects were examined in HepG2 cells and tumor-bearing mice, respectively. Morphological and release characterization showed that PTX/NCTD-APRPG-NPs were prepared successfully and achieved up to 90% release of PTX in a sustained manner. Compared with PTX/NCTD-NPs, PTX/NCTD-APRPG-NPs significantly enhanced the uptake of PTX. Notably, the inhibition of proliferation and migration of hepatoma cells was significantly higher in the PTX/NCTD-APRPG-NP group than those in the PTX-NP and PTX/NCTD-NP groups, which reflected significantly greater anti-tumor properties as well. Furthermore, key molecules in cell proliferation and apoptosis signaling pathways were altered most in the PTX/NCTD-APRPG-NP group, compared with the PTX-NP and PTX/NCTD-NP groups. Collectively, PTX/NCTD-loaded core–shell lipid nanoparticles modified with APRPG enhance the effectiveness of anti-HCC drugs and may be an effective system for the delivery of anti-HCC drugs.

## Introduction

Hepatocellular carcinoma (HCC) is the most common malignancy of the liver and a leading cause of cancer-related death worldwide (1). The majority of HCC cases occur in Asian countries and account for approximately 50% of all the new HCC cases globally (2, 3). Paclitaxel (PTX) is a naturally occurring anti-tumor agent that is isolated from the bark of *Taxus brevifolia.* PTX has been approved as a chemotherapeutic drug and is widely used as first-line treatment of a broad spectrum of cancers, including HCC, breast cancer, metastatic ovarian cancer, and malignant melanoma (4–7). PTX exerts its therapeutic effects through multiple effects, including inducing cell growth arrest at the G2/M phase, apoptosis, and autophagy, and proapoptotic death receptor (DR) 4 and DR5 have been shown to play a role in its mechanisms of action (6, 7). However, a number of limitations for PTX have been reported, such as its poor aqueous solubility, and PTX is usually formulated with solubilizing agents [Cremophor EL (CrEL) and ethanol] for clinical usage to avoid these limitations. However, CrEL in the PTX formulation has been reported to result in toxic effects, such as severe anaphylaxis, a potentially life-threatening allergy (8, 9). Therefore, research efforts have been made to develop novel formulations using agents other than CrEL, such as nanospheres (10), liposomes (11), nano-emulsions (12), and solid lipid nanoparticles (13), to prolong the *in vivo* half-life and residence time, and reduce the dosage and dosing frequency.

Nanoparticles have shown promising application in the delivery of anti-cancer drugs, including anti-HCC drugs (14, 15). Rapid progress has been made in the development of various nanoparticles, such as polymer, lipid, and metal nanoparticles, for the treatment of HCC (15). The use of nanoparticles has been shown to improve the targeting and therapeutic efficacy of anti-HCC drugs (14, 15). Nanoparticle drug delivery systems have advantages over traditional drug delivery methods, including a prolonged *in vivo* half-life and reduced systemic toxicity. Currently, liposome-based, polymer-based, and micelle-like nanoparticles are commonly used nanoparticle delivery systems for the treatment of malignant tumors (16, 17). However, these conventional nanoparticle-based delivery systems are clinically unsatisfactory (18–20).

Core–shell lipid nanoparticles have recently emerged as a new type of nanoparticle for drug delivery and show several beneficial characteristics, such as core–shell structure and programmable drug release (20). In the core–shell lipid nanoparticle delivery system, nanoparticles loaded with a drug in the core can be co-loaded with another drug in the phospholipid shell to form a more stable delivery system. Considering the advantages, such as controlled drug release, modifiable surface structure, and excellent biocompatibility, the core–shell lipid nanoparticle delivery system has become a research hotspot in the field of pharmaceutics (21). Notably, the loading of one drug in the core and the co-loading of another drug in the shell in the core–shell lipid nanoparticle delivery system enhances the therapeutic efficacy of the drugs compared with the conventional nanoparticle delivery systems. To date, anti-tumor drugs delivered using core–shell lipid nanoparticle delivery systems have not been explored for the treatment of HCC.

Norcantharidin (NCTD) is a demethylated derivative of cantharidin, an anti-cancer active ingredient of traditional Chinese medicine (22). Although NCTD has been shown as a promising anti-tumor oral agent for the treatment of various malignancies, the exact mechanism of action of NCTD remains unknown (22). However, the narrow therapeutic window and high renal toxicity have largely limited its clinical application (23). Therefore, it is urgent to develop novel formulations to improve the therapeutic effects and reduce the renal distribution of NCTD. Ala-Pro-Arg-Pro-Gly (APRPG) is a synthetic peptide that specifically targets tumor angiogenesis and was recently applied as a targeting peptide loaded in liposomes (24, 25).

In the present study, we prepared novel core–shell lipid nanoparticles loaded with PTX in the core as well as NCTD in the shell and modified with APRPG and investigated their anti-tumor effects in HCC.

## Materials and methods

### Preparation of PTX/NCTD-loaded core–shell lipid nanoparticles modified with APRPG (PTX/NCTD-APRPG-NPs)

PTX/NCTD-loaded core–shell lipid nanoparticles modified with APRPG (PTX/NCTD-APRPG-NPs) were prepared as described (20, 21). Briefly, we determined the optimal concentration of phospholipid as follows. We mixed 100 mg of cholesterol, 10 mg of mannose glycolipid, 10 mg of APRPG-PEG2000-DSPE, and 30 mg of NCTD in a flask and different concentrations of phospholipid (10 mg/ml, 20 mg/ml, and 30 mg/ml) were added. The phospholipid used in the formulation was lecithin (Shanghai Yuanye Bio-Technology Co., Ltd, Shanghai, China). The mixture was placed in a round-bottom flask in a rotary evaporator, 20 ml of chloroform was added, and the flask was placed in a 37°C water bath until the mixture was completely dissolved. After drying the remaining chloroform, the samples were dissolved in 10 ml of hexyl hydride. The sample was subjected to centrifugation; the supernatant was collected and the organic solvents were dried. The remaining mixture was dissolved in chloroform and a film was formed using a rotary evaporator. An appropriate volume of the PTX-PLGA nanoparticle suspension was added to the film, followed by hydration for 12 h. Finally, PTX/NCTD-APRPG-NPs were obtained. The particle size and uniformity of the film were measured to determine the optimal concentration of phospholipid. The minimal particle size and uniform film formation were observed in the 10 mg/ml phospholipid group. Therefore, a concentration of 10 mg/ml was used as the optimal phospholipid concentration in subsequent experiments.

The optimal ratio of phospholipid to cholesterol was determined from screening different ratios (1:1, 1:2, 2:1, 4:3, and 3:4) using the optimal phospholipid concentration of 10 mg/ml. The minimal particle size and uniform film formation were found in the 4:3 ratio group, and this was considered the optimal ratio of phospholipid to cholesterol.

After the optimal conditions were determined, we prepared PTX/NCTD-APRPG-NPs by dissolving 200 mg of phospholipid, 150 mg of cholesterol, 30 mg of NCTD, 10 mg of mannose glycolipid, and 10 mg of APRPG-PEG2000-DSPE in 20 ml of chloroform following the procedures described above.

For a comparative study, we also prepared core–shell lipid nanoparticles loaded with PTX/NCTD without 10 mg of APRPG-PEG2000-DSPE (PTX/NCTD-NPs) using similar procedures as described above. In addition, conventional PTX-loaded poly(lactic-co-glycolic acid) (PLGA) nanoparticles (PTX-NPs) with a core but without a shell structure were prepared as described previously (26).

The particle size was measured using the Beckman particle size analyzer (Beckman, CA, USA). The micromorphology of the APRPG-NPs and nanoparticles was observed by transmission electron microscopy. The encapsulation efficiency (EE) of the APRPG-NPs and nanoparticles was calculated after determining the PTX concentration in the APRPG-NPs and nanoparticles using high-performance liquid chromatography (HPLC). The APRPG-NPs and nanoparticles were placed under 4°C, 37°C, and 25°C for 24 h, and changes in the particle size were observed using the Beckman particle size analyzer (Beckman).

### *In vitro* release determination of PTX/NCTD-APRPG-NPs

Phosphate-buffered saline (PBS) containing 1% Tween 80 at pH 7.4 was used as the release medium. A 2-ml suspension of PTX/NCTD-APRPG-NPs was carefully transferred into a dialysis bag and placed into 20 ml of the release medium. The *in vitro* release assay was performed at 37°C with 100 shocks per minute. Three milliliters of media was collected at certain time points with supplementation of fresh media. The collected media was dried and the dried samples were dissolved in 1 ml of methyl alcohol, followed by filtration using a 0.22-μm Millipore filter. HPLC was used to detect the concentration of PTX and mass spectrometry (MS) was used to determine the concentration of NCTD in the samples to calculate the cumulative release percentage and generate the release curve as reported previously (27).

### Cell culture and cellular uptake assay

HepG2 cells were purchased from the American Type Culture Collection (ATCC) (Manassas, VA, USA) and cultured in Dulbecco’s Modified Eagle Medium (DMEM) supplemented with 10% fetal bovine serum (FBS) at 37°C and 5% CO_2_.

A cellular uptake assay was performed with HepG2 cells. The water-insoluble fluorescent dye coumarin 6 (COU6) used in the cellular uptake assay was purchased from Sigma-Aldrich (St Louis, MO, USA). Briefly, cells were incubated with COU6 (10 mg/ml) alone, COU6 (10 mg/ml) in combination with nanoparticles (COU6-nano), or COU6 (10 mg/ml) combined with PTX/NCTD-APRPG-NPs. After incubation for 2 h and washing three times with PBS buffer, the cells were visualized under an inverted fluorescence microscope (Olympus, Tokyo, Japan) at 37°C. The cellular uptake mechanism was investigated with a blocking experiment using free APRPG (10 mg/ml) and a low temperature test was conducted at 4°C.

### Cell proliferation assay

The 3-[4,5-dimethylthiazol-2-yl]-2,5 diphenyl tetrazolium bromide (MTT) assay was conducted to assess cell proliferation. In brief, HepG2 cells were seeded in 96-well plates at a density of 5×10^3^ cells/well in 200 μl of DMEM supplemented with 10% FBS and incubated with 200 μl of MTT solution (1 mg/ml) for 4 h. After removing the supernatant, 150 μl of dimethyl sulfoxide (DMSO) was added to solubilize the MTT-formazan crystals. Following incubation for 15 min, the absorbance at 570 nm was read on a Microplate Reader (PerkinElmer, MA, USA).

### Transwell assay

Transwell assays were performed using a Transwell kit (BD Bioscience, CA, USA). Briefly, HepG2 cells (8×10^4^ cells) were suspended in 300 μl of serum-free DMEM medium and placed in the upper chamber of the insert; complete medium was added into the lower chamber. After incubation for 36 h, the cells were fixed with methanol and stained with Giemsa; the cells on the top surface of the membrane were wiped off and the cells on the lower surface were analyzed under a microscope (Olympus DP72, Olympus, Tokyo, Japan). The average number of migrated cells was determined to measure the migration capacity.

### Animals

Female BALB/c nude mice (4–6 weeks of age, 16–20 g) were purchased from Shanghai Slac Laboratory Animal Co. Ltd. (Shanghai, China) [animal certificate: SCXK (Shanghai) 2007-0005]. Experimental mice were housed in a specific-pathogen-free (SPF) animal facility at the Animal Experimental Center, Zhejiang University (Hangzhou, Zhejiang, China) and maintained under controlled conditions: room temperature (22°C), SPF conditions, and a 12/12-h light/dark cycle with free access to normal chow and water. The protocols involving the use of experimental animals were approved by the Laboratory Animal Care and the Department of Laboratory Animal Research of Zhejiang University.

### Tumor model and tissue distribution assay

A total of 27 BALB/c nude mice were used to generate a tumor-bearing model for an *in vivo* tissue distribution assay. BALB/c nude mice were injected subcutaneously with 5×10^6^ HepG2 cells to establish a tumor-bearing mouse model.

After 7 days of implantation, when the tumor volume reached approximately 500 mm^3^, the mice were injected through the tail vein with PTX-NPs, PTX/NCTD-NPs, and PTX/NCTD-APRPG-NPs [in normal saline (NC)] at a dose of 10 mg/kg (PTX). The mice were euthanized at 0.5, 2, and 6 h post-injection (*n* = 9 for each time point). Heart, lung, spleen, stomach, kidney, and tumor were collected and washed with NC, followed by homogenization. The concentration of PTX and NCTD in the tissues was measured using HPLC coupled with electrospray ionization MS (LC-MS) (**Supplementary Figures 1, 2**). The conditions for HPLC were as follows: Agilent ZORBAX HPLC analytic column-C18 (3.0×100 mm, 1.7 μm particle diameter); column temperature, 40°**C;** mobile phase composition (mobile phase A, pure water; mobile phase B, acetonitrile solution); wavelength (210 nm, 227 nm) for HPLC UV detector; flow rate, 0.4 ml/min; and an injection volume of 10 μl (28, 29). The conditions for MS were as follows: mass spectrometer (Xevo G2-XS QTOF, Waters, USA); ion generation, electrospray ionization (ESI); quantification, multiple reaction monitoring (MRM) mode; scan mode, negative ion mode; capillary voltage, 2 kV; cone voltage, 40 V; scan time, 0.2 s; data acquisition mode, MSE.

### *In vivo* imaging for assessment of tissue distribution

The distribution of PTX was visualized with a Bruker Small Animal Optical Imaging System (*In Vivo* Xtreme II, MA, USA). The PTX/NCTD-NPs and PTX/NCTD-APRPG-NPs were prepared with fluorescein isothiocyanate (FITC)-PTX (Sigma, New York, NY, USA) according to the manufacturer’s instructions. BALB/c nude mice were injected subcutaneously with 5×10^6^ HepG2 cells. After 7 days, when the tumor volume reached 500 mm^3^, the mice were injected through the tail vein with FITC-PTX-NPs, FITC-PTX/NCTD-NPs (prepared with FITC-PTX), and PTX/NCTD-APRPG-NPs (prepared with FITC-PTX). The tissue distributions of PTX were visualized and imaged on the Bruker Small Animal Optical Imaging System (*In‐Vivo* Xtreme II; MA, USA) at 2 h post-injection.

### Examination of anti-tumor effects in mice

To examine the *in vivo* anti-tumor effects of the NPs, 27 BALB/c nude mice were injected subcutaneously with 5×10^6^ HepG2 cells. After the tumor volume reached approximately 200 mm^3^, the mice were injected through the tail vein with NC (as the control), PTX-NPs, PTX/NCTD-NPs, or PTX/NCTD-APRPG-NPs at a dosage of 6 mg/kg (PTX) on days 1, 4, 7, 10, 13, and 16. Tumor volume and body weights were measured on days 1, 4, 7, 10, 13, and 16. Upon completion of the experiments, the mice were euthanized and the tumors were collected and weighted. The inhibitory rate was calculated in each group.

### Flow cytometry

Flow cytometry was performed to examine the effects of PTX-NPs, PTX/NCTD-NPs, and PTX/NCTD-APRPG-NPs on apoptosis and the cell cycle distribution in HepG2 cells. Apoptotic cells were determined using an FITC Annexin V Apoptosis Detection Kit I (BD Pharmingen, CA, USA) according to the manufacturer’s instructions. Briefly, 5 μl of propidium iodide (PI) and 5 μl of Annexin V were added to HepG2 cells (1×10^6^ cells) and cells were incubated at room temperature for 30 min. For cell cycle analysis, cellular DNA content was examined following cell staining with PI. The percentages of cell populations in different phases of the cell cycle (G0/G1, S, and G2/M) were assayed on the BD flow cytometer (BD Pharmingen, CA, USA) following the manufacturer’s protocol.

### Western blot analysis

Total proteins were isolated from treated HeG2 cells using radioimmunoprecipitation assay (RIPA) Lysis Buffer (Beyotime, Shanghai, China) and protein concentrations were quantified with a BCA kit (Beyotime). Protein samples were separated by 12% sodium dodecyl sulfate polyacrylamide gel electrophoresis (SDS-PAGE) and transferred to polyvinylidene difluoride (PVDF) membranes. After blocking with 5% bovine serum albumin (BSA), the membrane was incubated with primary antibodies against phosphorylated protein kinase B (p-AKT; 1:1,000, CST, Boston, MA, USA), AKT (1:1,000, CST), phosphorylated extracellular signal-regulated protein kinase (p-ERK1/2; 1:1,000, CST), ERK1/2 (1:1,000, CST), phosphorylated mitogen-activated protein kinase kinase (p-MEK; 1:1,000, CST), MEK (1:1,000, CST), caspase-3 (1:1,000, CST), cleaved caspase-3 (1:1,000, CST), B-cell lymphoma 2 (Bcl-2; 1:1,000, CST), and glyceraldehyde 3-phosphate dehydrogenase (GAPDH; 1:1,000, CST) at 4°C overnight. The membranes were then incubated with secondary antibody at room temperature for 1.5 h. Protein bands were visualized using ECL solution (Beyotime, Shanghai, China) on the Tanon 5200-multi (Tanon, Shanghai, China). Quantification was performed using ImageJ software to determine the relative expression level of the target proteins.

### Statistical analysis

Data are expressed as the means ± standard deviation (SD) of at least three independent experiments. The unpaired Student’s *t*-test was used to compare quantitative data between two study groups when the groups were not matched. One-way analysis of variance (ANOVA) followed by Tukey’s test for *post-hoc* analysis was used to determine statistical differences between the mean values for multiple groups. *p*-values  lower than  0.05 were considered to be statistically significant.

## Results

### Preparation and characterization of PTX-NPs, PTX/NCTD-NPs, and PTX/NCTD-APRPG-NPs

**Figure 1:**
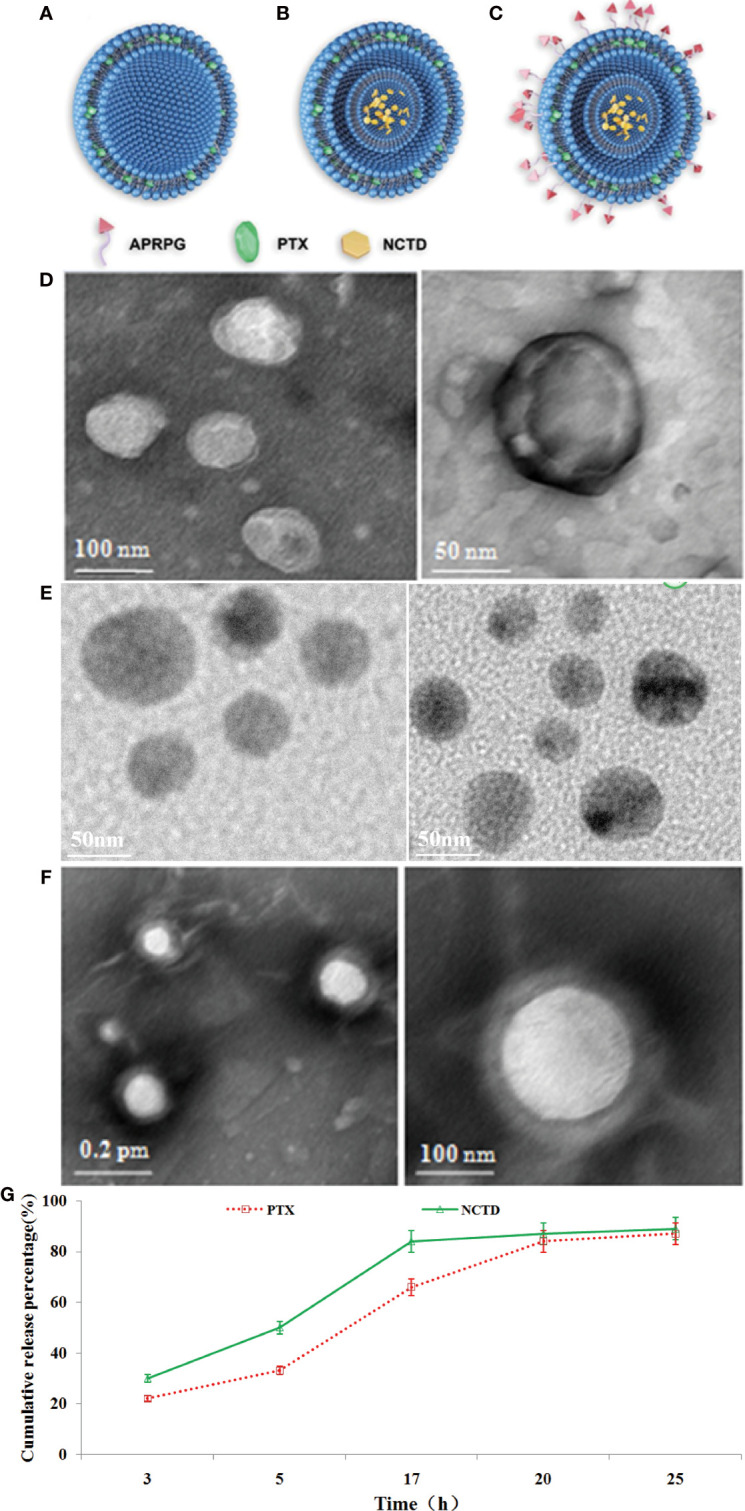
The structural, morphological, and release characteristics of PTX-NPs and PTX/NCTD-APRPG-NPs. Schematic illustration of the structure and composition of nanoparticles used in this study, including **(A)** PTX-loaded PLGA nanoparticles (PTX-NPs); **(B)** PTX/NCTD-loaded core–shell-type nanoparticles (PTX/NCTD-NPs); and **(C)** PTX/NCTD-loaded core–shell lipid nanoparticles modified with APRPG (PTX/NCTD-APRPG-NPs). The morphological characteristics of PTX-NPs and PTX/NCTD-APRPG-NPs, including the shape and size, were examined with transmission electron microscopy (TEM) and the Zetasizer instrument, respectively. **(D)** The shape of the PTX-NPs; **(E)** The shape of the PTX/NCTD-NPs; **(F)** The shape of the PTX/NCTD-APRPG-NPs; **(G)** *in vitro* release characteristic of PTX/NCTD-APRPG-NPs. PTX, paclitaxel; PLGA, poly(lactic-co-glycolic acid); NPs, nanoparticles; NCTD, norcantharidin; APRPG, tumor neovasculature-targeted peptide (Ala-Pro-Arg-Pro-Gly).

We prepared PTX-loaded PLGA nanoparticles (PTX-NPs), PTX/NCTD-loaded core–shell-type nanoparticles (PTX/NCTD-NPs), and PTX/NCTD-loaded core–shell-type nanoparticles modified with APRPG (PTX/NCTD-APRPG-NPs). The structure and composition of PTX-NPs, PTX/NCTD-NPs, and PTX/NCTD-APRPG-NPs are schematically illustrated in **Figures 1A–C**. Their morphological and release characteristics are shown in **Figures 1D, E**. The PTX-NPs were morphologically spherical and uniform in shape and size (~100 nm). Similarly, the PTX/NCTD-APRPG-NPs presented a spherical shape and size of around 100 nm (**Figures 1F, G**). We evaluated the *in vitro* release characteristics of the PTX/NCTD-APRPG-NPs, and the findings are presented in **Figure 1H**. Notably, up to 85% of PTX/NCTD was released into the cell culture medium within 18–20 h in a sustained manner.

### PTX/NCTD-APRPG-NPs enhanced the inhibitory effects of PTX on the proliferation and migration of hepatoma cells

**Figure 2:**
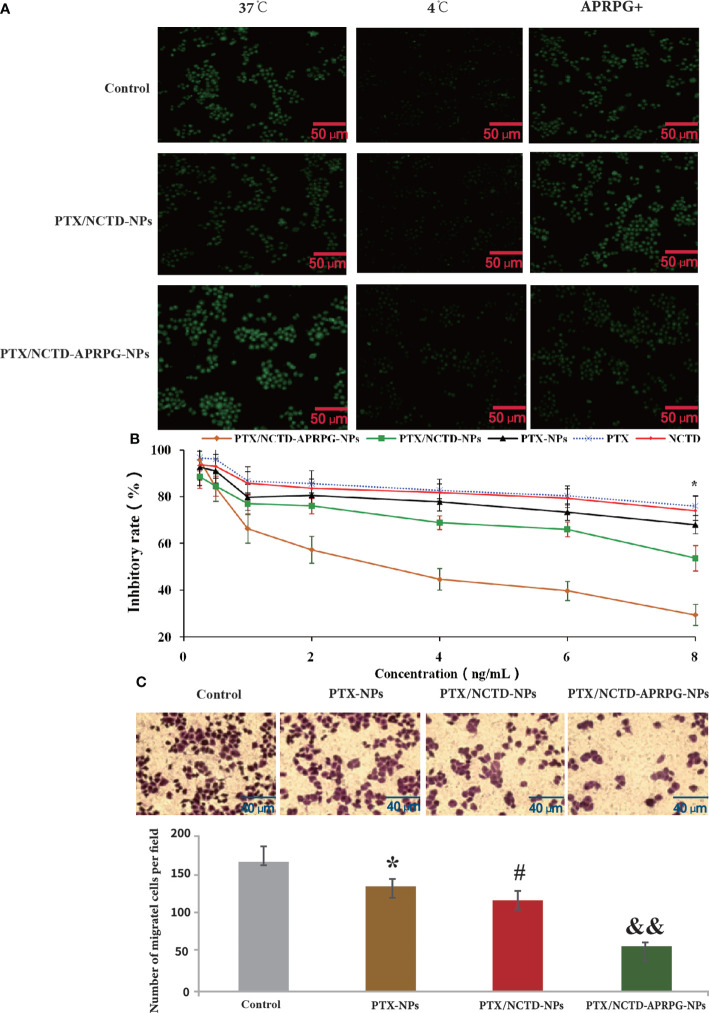
*In vitro* uptake and anti-tumor effects of PTX-NPs, PTX/NCTD-NPs, and PTX/NCTD-APRPG-NPs. HepG2 cells were treated with PTX-NPs, PTX/NCTD-NPs, or PTX/NCTD-APRPG-NPs, and the proliferation and migration abilities were assayed using MTT and Transwell assays, respectively. A fluorescence assay was performed to examine the uptake of PTX. **(A)** Fluorescence microscopic images showing nanoparticle uptake in HepG2 cells. HepG2 cells were incubated with COU6 (10 mg/ml) alone, COU6 (10 mg/ml) in combination with nanoparticles (COU6-nano), or COU6-nano modified with APRPG (APRPG-COU6-nano). After incubation for 2 h and washing three times with PBS buffer, cells were visualized under a fluorescence microscope. **(B)** Comparison of the effects of PTX/NCTD-NPs and PTX/NCTD-APRPG-NPs on the proliferation of HepG2 cells. HepG2 cells were treated with PTX/NCTD-APRPG-NPs, PTX/NCTD-NPs, PTX-NPs, PTX, or NCTD, and proliferation was assayed using MTT; *p < 0.001. **(C)** Effects of PTX-NPs, PTX/NCTD-NPs, and PTX/NCTD-APRPG-NPs on the migration ability of HepG2 cells. HepG2 cells were treated with PTX-NPs, PTX/NCTD-NPs, or PTX/NCTD-APRPG-NPs, and migration ability was examined using Transwell assays. *p < 0.05 vs. control; #*p* < 0.05 vs. PTX; &&*p* < 0.01 vs. nanoparticles.

Before determining the anti-tumor effects of the PTX/NCTD-APRPG-NPs, we first examined the uptake of APRPG-modified nanoparticles in HepG2 cells. As shown in **Figure 2A** and **Supplementary Figure 3**, using an incubation temperature of 4°C as the negative control, the fluorescence intensity at 37°C in the APRPG-COU6-nano group was significantly higher than that in the COU6 only and COU6-nano groups (*p* < 0.05). Moreover, when cells were incubated with APRPG (10 mg/ml) before the COU6 formulations, the intracellular fluorescence intensity of APRPG-COU6-nano was substantially reduced, whereas the fluorescence intensity in the COU6 and COU6-nano groups remained unchanged (**Figure 1D** and **Supplementary Figure 3**). The difference may be attributed to the specific binding of APRPG in HepG2 cells. Free APRPG peptide competitively combined with the ligands instead of APRPG-COU6-nano.

We further investigated the effects of PTX/NCTD-APRPG-NPs on the proliferation of human hepatocytes (L0 cells) and hepatoma cells (HepG2, Huh-7, and Hep3B cells). As shown in **Figure 2B**, PTX/NCTD-APRPG-NPs exhibited significantly greater inhibitory effects than PTX/NCTD-NPs, with half-maximal inhibitory concentration (IC_50_) values of 4.173 ng/ml versus 7.976 ng/ml against HepG2 cells, 4.973 ng/ml versus 9.264 ng/ml against Huh-7 cells, and 6.933 ng/ml versus 13.06 ng/ml against Hep3B cells (all *p* < 0.05). In addition, analysis of hepatoma cell migration capabilities showed that the number of migrated cells was significantly suppressed in the PTX-NP, PTX-NCTD-NP, and PTX/NCTD-APRPG-NP groups compared with the number in the control groups (**Figure 2C**). The PTX/NCTD-APRPG-NP group had the lowest number of migrated cells (*p* < 0.05 vs. control, *p* < 0.05 vs. PTX-NPs, *p* < 0.01 vs. PTX/NCTD-NPs). These data indicated that the PTX/NCTD-APRPG-NPs enhanced the inhibitory effects of the drugs on the proliferation and migration of hepatoma cells compared with the PTX-NPs and PTX/NCTD-NPs.

### PTX/NCTD-APRPG-NPs exerted significant anti-tumor effects in mice

**Figure 3:**
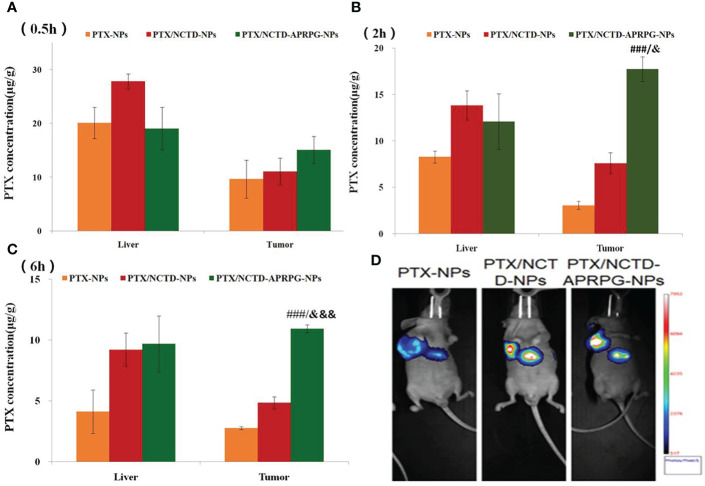
*In vivo* distribution of PTX in tumor-bearing mice treated with PTX-NPs, PTX/NCTD-NPs, and PTX/NCTD-APRPG-NPs. The experimental mice were randomly divided into three groups (PTX-NP, PTX/NCTD-NP, and PTX/NCTD-APRPG-NP groups) and injected with PTX-NPs, PTX/NCTD-NPs, or PTX/NCTD-APRPG-NPs at the dosage of 10 mg/kg. The tissue distribution of PTX was detected at 0.5, 2, and 6 h following injection. Bar graphs show PTX concentrations in various organs (heart, liver, spleen, lung, kidney, and stomach) and tumor tissues at **(A)** 0.5 h, **(B)** 2 h, and **(C)** 6 h after injection; ###*p* < 0.001 vs. PTX-NPs; &*p* < 0.05, &&&*p* < 0.001 vs. PTX/NCTD-NPs. **(D)** *In vivo* imaging assay to visualize the distribution of PTX in tumor-bearing mice treated with PTX-NPs, PTX/NCTD-NPs, and PTX/NCTD-APRPG-NPs. At 2 h after injection, the tissue distributions of PTX in the three experimental groups were imaged on the Bruker Small Animal Optical Imaging System.

Based on the *in vitro* studies, we further investigated the anti-tumor effects of PTX/NCTD-APRPG-NPs in tumor-bearing nude mice. The tissue distribution and anti-tumor effects of PTX-NPs, PTX/NCTD-NPs, and PTX/NCTD-APRPG-NPs are illustrated in **Figure 3**. At the three examined time points, the concentration of PTX in the tumor tissues was in the following order: PTX/NCTD-APRPG-NPs > PTX/NCTD-NPs > PTX-NPs, indicating that the PTX/NCTD-APRPG-NPs had a better targeting effect than the PTX/NCTD-NPs and PTX-NPs. The tissue distribution of the injected PTX/NCTD-APRPG-NPs and PTX/NCTD-NPs was further confirmed by *in vivo* imaging. As shown in **Figure 3D**, higher fluorescence intensity was observed in the tumor tissues and lower fluorescence intensity was detected in the liver tissues of PTX/NCTD-APRPG-NP-treated animals compared with the PTX/NCTD-NP group, indicating that PTX/NCTD-APRPG-NPs had potential lower toxicity and higher tumor-targeting characteristics.

**Figure 4:**
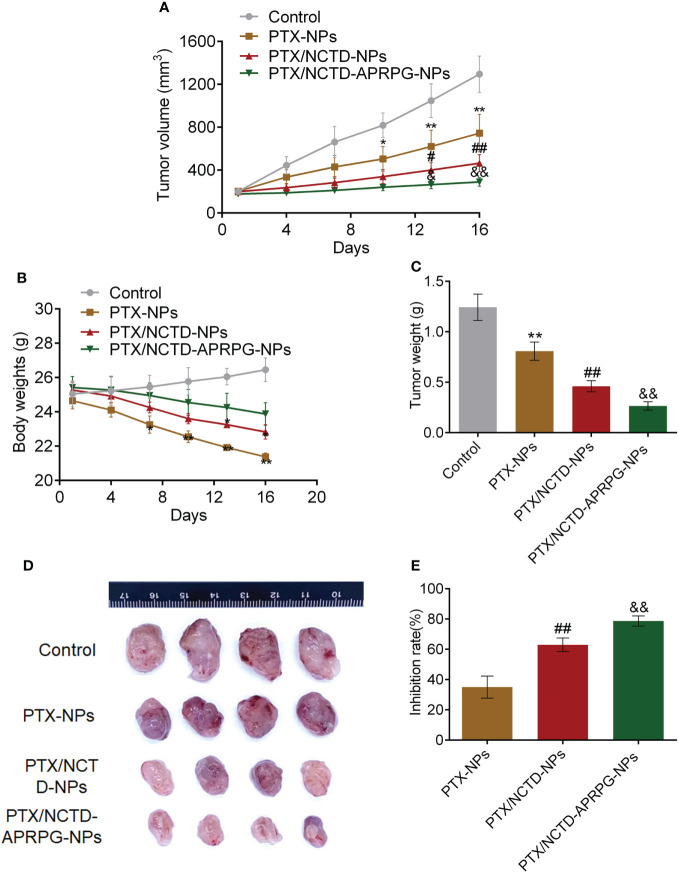
*In vivo* anti-tumor effects of PTX-NPs, PTX/NCTD-NPs, and PTX/NCTD-APRPG-NPs in the HepG2 xenograft mouse model. **(A)** Effects of PTX-NPs, PTX/NCTD-NPs, and PTX/NCTD-APRPG-NPs on the tumor volume at different time points (days 1, 4, 7, 10, 13, and 16 after the first administration); **(B)** effects of PTX-NPs, PTX/NCTD-NPs, and PTX/NCTD-APRPG-NPs on the body weights at different time points (days 1, 4, 7, 10, 13, and 16 after the first administration); **(C)** effects of PTX-NPs, PTX/NCTD-NPs, and PTX/NCTD-APRPG-NPs on the tumor weight on day 16 after the first administration; **(D)** effects of PTX-NPs, PTX/NCTD-NPs, and PTX/NCTD-APRPG-NPs on the tumor size; and **(E)** effects of PTX-NPs, PTX/NCTD-NPs, and PTX/NCTD-APRPG-NPs on tumor growth in the HepG2 xenograft animal model. *p < 0.05 vs. control, **p < 0.01 vs. control, #*p* < 0.05 vs. PTX-NPs, ##*p* < 0.01 vs. PTX-NPs, &*p* < 0.05 vs. PTX/NCTD-NPs, &&*p* < 0.01 vs. PTX/NCTD-NPs.

The anti-tumor effects and toxicity are shown in **Figure 4** and **Supplementary Figure 4**. The tumor volume was suppressed to the greatest extent by PTX/NCTD-APRPG-NPs, followed by PTX/NCTD-NPs and PTX-NPs (*p* < 0.05 vs. control, *p* < 0.01 vs. control, *p* < 0.05 vs. PTX-NPs, *p* < 0.01 vs. PTX-NPs, *p* < 0.05 vs. PTX/NCTD-NPs, *p* < 0.01 vs. PTX/NCTD-NPs) (**Figure 4A**). As shown in **Figure 4B**, the body weights were slightly lower in the PTX/NCTD-APRPG-NP group, followed by the PTX/NCTD-NP and PTX-NP groups (*p* < 0.05 vs. control, *p* < 0.01 vs. control). The tumor weight was significantly lower in the PTX-NP group compared with controls and was dramatically further inhibited in the PTX/NCTD-NP group (**Figure 4C**). A pronounced decrease in the tumor weight was observed in the PTX/NCTD-APRPG-NP group compared with the PTX/NCTD-NP group (*p* < 0.01 vs. control, *p* < 0.01 vs. PTX-NPs, *p* < 0.01 vs. PTX/NCTD-NPs). As shown in **Figures 4D, E**, the PTX/NCTD-APRPG-NPs exhibited the greatest anti-tumor effects, with the smallest tumor size in the PTX/NCTD-APRPG-NP group (**Figure 4D**) and highest inhibitory rate of 78.67%. The inhibitory rate was 62.98% in the PTX/NCTD-NP group and 35.01% in the PTX-NP group (*p* < 0.01 vs. PTX-NPs, *p* < 0.01 vs. PTX/NCTD-NPs). Histological examinations of tumor tissues further revealed the greatest anti-tumor effect of PTX/NCTD-APRPG-NPs compared with the other treatments (**Supplementary Figure 4**).

### PTX/NCTD-APRPG-NPs induced apoptosis through the AKT and ERK pathways

**Figure 5:**
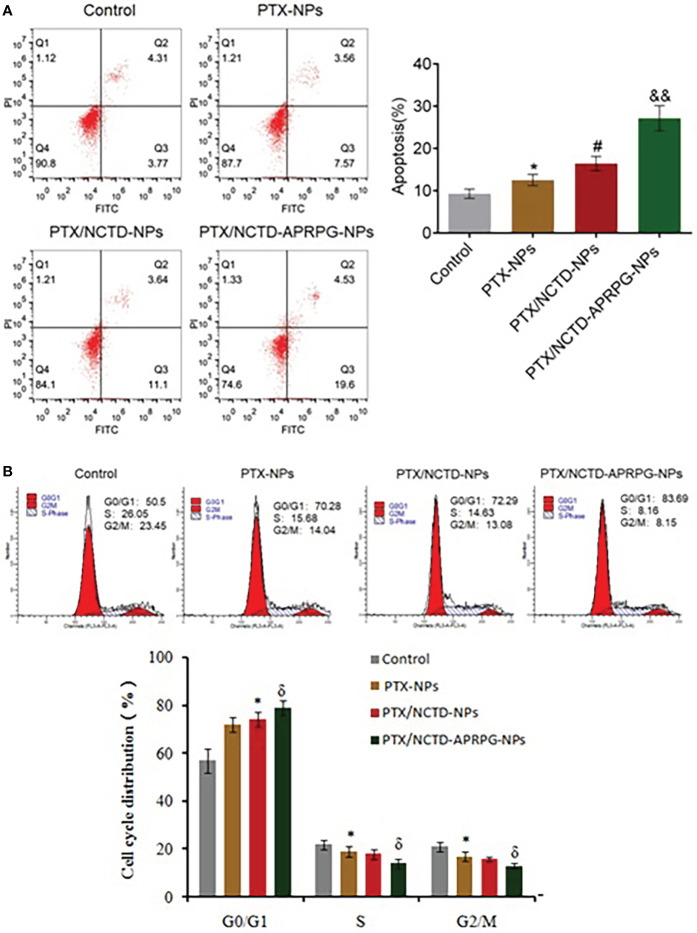
Apoptosis and cell cycle arrest were induced by PTX/NCTD-APRPG-NPs. Flow cytometry was conducted to determine the number of apoptotic cells and cell cycle distribution in HepG2 cells treated with PTX-NPs, PTX/NCTD-NPs, and PTX/NCTD-APRPG-NPs. **(A)** Effects of PTX-NPs, PTX/NCTD-NPs, and PTX/NCTD-APRPG-NPs on apoptotic rates in HepG2 cells; **(B)** effects of PTX-NPs, PTX/NCTD-NPs, and PTX/NCTD-APRPG-NPs on the cell cycle distribution in HepG2 cells. *p < 0.05 vs. control, #*p* < 0.05 vs. PTX-NPs, &*p* < 0.05 vs. PTX/NCTD-NPs, &&*p* < 0.01 vs. PTX/NCTD-NPs.

To investigate whether the PTX/NCTD-APRPG-NPs exerted the anti-tumor effects through targeting apoptosis, we compared apoptotic rates between groups and examined key molecules related to apoptosis in HepG2 cells. As shown in **Figure 5**, the apoptotic rate was 24.13% in the PTX/NCTD/APRPG-NP group, which was significantly higher than 14.74% in the PTX/NCTD-NP group, 11.13% in the PTX-NP group, and 8.08% in the control group (*p* < 0.05 vs. control, *p* < 0.05 vs. PTX-NPs, and *p* < 0.01 vs. PTX/NCTD-NPs). In addition, the proportion of cells arrested at the G0/G1 phase was 70.28% in the PTX-NP group, which was significantly greater than 50.5% in the control group (*p* < 0.05 vs. control). Notably, the proportion of cells arrested at the G0/G1 phase in the PTX/NCTD-APRPG-NP group was 83.69%, which was significantly greater than 72.29% in the PTX/NCTD-NP group (*p* < 0.05 vs. PTX/NCTD-NPs). These data indicated that apoptosis and cell cycle arrest at the G0/G1 phase were induced to a significantly greater extent by the PTX/NCTD-APRPG-NPs than by the PTX/NCTD-NPs or PTX-NPs.

**Figure 6:**
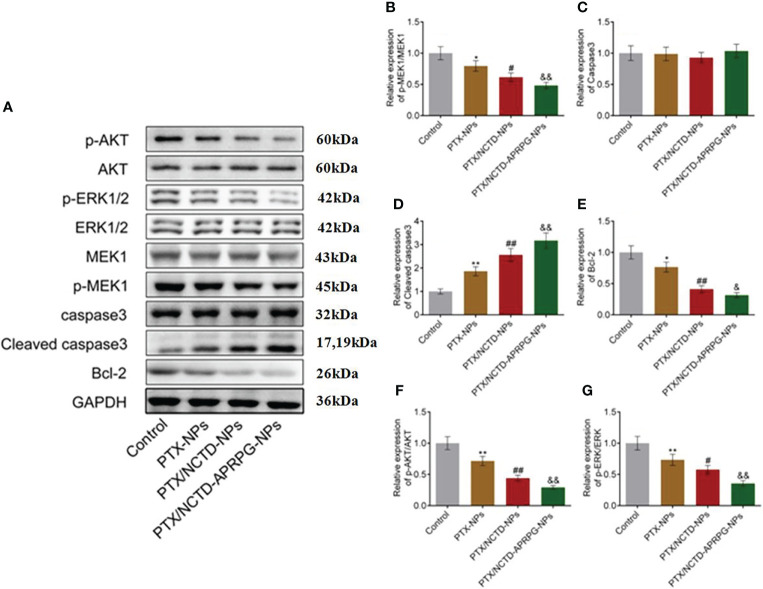
Effects of PTX-NPs, PTX/NCTD-NPs, and PTX/NCTD-APRPG-NPs on the AKT and ERK signal pathways in HepG2 cells. Western blot analysis was performed on AKT and ERK pathway protein expression in HepG2 cells treated with PTX-NPs, PTX/NCTD-NPs, and PTX/NCTD-APRPG-NPs. **(A)** Western blot images; **(B)** quantitative analysis of relative expression of p-MEK1/MEK1; **(C)** relative expression of caspase-3; **(D)** relative expression of cleaved caspase-3; **(E)** relative expression of Bcl-2; **(F)** relative expression of pAKT/AKT; and **(G)** relative expression of p-ERK/ERK. *p < 0.05 vs. control, **p < 0.01 vs. control, #*p* < 0.05 vs. PTX-NPs, ##*p* < 0.01 vs. PTX-NPs, &*p* < 0.05 vs. PTX/NCTD-NPs, &&*p* < 0.01 vs. PTX/NCTD-NPs.

We further performed Western blot analysis to determine whether the AKT and ERK signaling pathways were involved in the PTX/NCTD-APRPG-NP-induced apoptosis. As shown in **Figure 6**, p-AKT, p-MEK, p-ERK1/2, and Bcl-2 protein expression was significantly suppressed by the introduction of PTX-NPs compared with the control group and was further inhibited by treatment with the PTX/NCTD-NPs. p-AKT, p-MEK, p-ERK1/2, and Bcl-2 protein expressions were markedly decreased by treatment with PTX/NCTD-APRPG-NPs (*p* < 0.05 vs. control, *p* < 0.01 vs. control, *p* < 0.05 vs. PTX-NPs, *p* < 0.01 vs. PTX-NPs, *p* < 0.05 vs. PTX/NCTD-NPs, and *p* < 0.01 vs. PTX/NCTD-NPs). In addition, PTX-NP-induced cleaved caspase-3 protein expression was significantly promoted by treatment with PTX/NCTD-NPs and further enhanced by PTX/NCTD-APRPG-NPs (*p* < 0.01 vs. control, *p* < 0.01 vs. PTX-NPs, *p* < 0.01 vs. PTX/NCTD-NPs). These findings, together with those of others, suggested that the AKT and ERK signal pathways were involved in apoptosis induced by PTX/NCTD-APRPG-NPs (30–32).

## Discussion

In this study, we developed new core–shell-type nanoparticles loaded with chemically distinct drugs PTX/NCTD and modified with APRPG and assessed the anti-tumor effects in hepatoma cells (HepG2, Huh-7, and Hep3B) and tumor-bearing nude mice. The novel findings of our study are summarized as follows (1): PTX/NCTD-APRPG-NPs with a core–shell structure were prepared successfully and achieved up to 90% release of PTX in a sustained manner; (2) PTX/NCTD-APRPG-NPs led to significantly greater anti-tumor effects in comparison with PTX-NPs and PTX/NCTD-NPs in both *in vitro* and *in vivo* studies; and (3) mechanistic studies revealed that PTX/NCTD-APRPG-NPs markedly induced apoptosis through the AKT and ERK signal pathways, and the effects of these nanoparticles were the greatest among the groups evaluated. These findings demonstrated that the PTX/NCTD-APRPG-NPs enhanced the effectiveness of anti-HCC drugs.

Core–shell lipid nanoparticles are a novel group of lipid/polymer particle assemblies composed of lipid shells and nanoparticle cores that possess the physical characteristics of both lipid vesicles and nanoparticles (33, 34). Recently, core–shell lipid nanoparticles have been applied in multiple biomedical fields, such as drug delivery and genetic transmission (35). Based on differences in the materials, especially those used in the core, core–shell lipid nanoparticles can be classified into four groups, of which silica (36), nanogel (37), polysaccharide (38), and polymer nanoparticles were used as the nanoparticle core (39). In the present study, PLGA was used as the nanoparticle core to load PTX. PLGA is derived from the polymerization between lactic acid and glycolic acid [poly(D,L-lactic-co-glycolic acid)]. PLGA has various advantages, including beneficial characteristics such as biocompatibility and degradation, which make PLGA the most widely used polymer in the design of nanoparticles (40). In the present study, the concentration of emulsifier, PLGA, PTX, and phospholipid; the shear velocity; and the ratio of phospholipid versus cholesterol were screened to obtain a minimal particle size and uniform film formation. According to the previous findings (41), we observed uniform spherical morphology, 100-nm particle size, and sustained release with the core–shell lipid nanoparticles prepared in this study.

Combination drug therapy using two or more drugs is an important application for core–shell lipid nanoparticles. Sengupta first reported the application of core–shell lipid nanoparticles in anti-tumor combination drug therapy (42). With combretastatin wrapped in the phospholipid shell and adriamycin wrapped in the nanoparticles, core–shell lipid nanoparticles were prepared with both promising anti-angiogenesis and cytotoxic properties, leading to more significant effects than the mixture of combretastatin and adriamycin. Wong (43) reported that core–shell lipid nanoparticles loaded with both the inhibitor of P-gp protein and adriamycin showed significant inhibitory effects on adriamycin-resistant breast cancer cell lines. In the present study, we prepared core–shell lipid nanoparticles loaded with PTX in the core and NCTD in the shell and compared the anti-tumor effects with those of PTX-NPs and PTX/NCTD-NPs. Notably, PTX/NCTD-APRPG-NPs showed more significant inhibitory effects on the proliferation and migration of hepatoma cells, as well as higher inhibitory rates on tumor growth in the HepG2 xenograft animal model in mice. These results demonstrate that the anti-tumor property of PTX was markedly enhanced by the combination of PTX and NCTD using core–shell lipid nanoparticles.

Notably, our results showed that PTX/NCTD-APRPG-NPs exhibited the greatest anti-tumor activity among the tested nanoparticles. APRPG is a short peptide that targets neovascularization and specifically targets the vascular endothelial growth factor receptor 1 (VEGFR-1), which is highly expressed in neovascularization tissues (44). Recent studies have reported the application of nanoliposomes modified with APRPG for targeting angiogenesis (45, 46). In the present study, APRPG was used to modify the core–shell lipid nanoparticles to enhance the tumor-targeting property. Compared with PTX/NCTD-NPs without APRPG, PTX/NCTD-APRPG-NPs showed more significant inhibitory effects on the proliferation and migration of hepatoma cells, as well as greater inhibitory effects on tumor growth in the HepG2 xenograft animal model in mice. These *in vitro* and *in vivo* findings indicated that the anti-tumor property of PTX/NCTD-NPs was dramatically elevated by the addition of APRPG in PTX/NCTD-APRPG-NPs. In addition, *in vitro* uptake assays and *in vivo* imaging revealed that the tumor targeting property of PTX/NCTD-NPs was significantly promoted by the modification with APRPG.

Previous reports have shown that PTX alone induces cell growth arrest at the G2/M phase (1–3). In the present study, we found that PTX-NPs, PTX/NCTD-NPs, and PTX/NCTD-APRPG-NPs markedly induced cell cycle arrest at the G0/G1 phase, which was different from the induction of cell cycle arrest at the G2/M phase by PTX alone. In addition, cell cycle arrest at the G0/G1 phase was significantly induced to a greater extent by the PTX/NCTD-APRPG-NPs than by the PTX/NCTD-NPs or PTX-NPs. Further mechanistic studies confirmed that the AKT and ERK signal pathways were involved in the PTX/NCTD-APRPG-NP-induced cell cycle arrest at the G0/G1 phase. This may be related to MEPK pathway, in which MEK and ERK pathways are all important branch pathways. Previous studies have found that MEK inhibitor can truly induce G1 cell cycle arrest in tumor cells. The ERK pathway is constitutively activated, which indicated that the ERK pathway might be a potential therapeutic target (47, 48). Moreover, there is an interesting observation that cell cycle arrest was induced at the G2/M phase by PTX alone and at the G0/G1 phase by PTX/NPs as well. Further in-depth mechanistic studies are needed to gain insight into the mechanism of action. In addition, further investigations, including *in vitro* angiogenesis assays (e.g., chick embryo chorioallantoic membrane assay, tube formation assay), are necessary to confirm the enhanced neovascularization targeting property of PTX/NCTD-APRPG-NPs and are underway in our laboratory.

Taken together, our study has demonstrated that APRPG-modified PTX/NCTD-loaded core–shell lipid nanoparticles markedly improved the anti-tumor effectiveness of anti-HCC drugs. Therefore, the newly developed PTX/NCTD-APRPG-NPs hold promise as a potent nanoparticle delivery system in the treatment of HCC.

## Data availability statement

The raw data supporting the conclusions of this article will be made available by the authors, without undue reservation.

## Ethics statement

The animal study was reviewed and approved by the Animal Laboratory Committee of Zhejiang University (Hangzhou, Zhejiang, China).

## Author contributions

M-HX: Experimental design, literature inquiry, prescription design and optimization, animal experiment, and data processing. Z-LF and A-LH: Literature inquiry and preparation of the drug delivery system. QC and J-BL: Animal experiment. J-FZ, SY, and LZ: Cell experiment. X-JC and MG: Preparation of the drug delivery system. JW: Experimental guidance. All authors contributed to manuscript revision, read, and approved the submitted version.

## Funding

This work was financially supported by the Science and Technology Project of Hangzhou City (Agriculture and Social Development, No. 2016007), the Science and Technology Project of Yuhang District, Hangzhou City (No. 2017002), the Project of Hangzhou City (Agriculture and Social Development, No. 20201231Y131), the Science and Technology Project of Yuhang District, Hangzhou City (2014003), the Science and Technology Project of Hangzhou City (Social Development) (20140633B57), and the Health Science and Technology Project of Hangzhou City (2015B32).

## Conflict of interest

The authors declare that the research was conducted in the absence of any commercial or financial relationships that could be construed as a potential conflict of interest.

## Publisher’s note

All claims expressed in this article are solely those of the authors and do not necessarily represent those of their affiliated organizations, or those of the publisher, the editors and the reviewers. Any product that may be evaluated in this article, or claim that may be made by its manufacturer, is not guaranteed or endorsed by the publisher.

## Glossary

**Table:**

APRPG	Ala-Pro-Arg-Pro-Gly
ATCC	American Type Culture Collection
COU6	coumarin 6
DMEM	Dulbecco's Modified Eagle Medium
DMSO	dimethyl sulfoxide
EE	encapsulation efficiency
FBS	fetal bovine serum
FITC	fluorescein isothiocyanate
HCC	hepatocellular carcinoma
HPLC	high-performance liquid chromatography
MTT	3-[4,5-dimethylthiazol-2-yl]-2, 5 diphenyl tetrazolium bromide
NC	normal saline
NCTD	norcantharidin
PBS	Phosphate-buffered saline
PLGA	poly (lactic-co-glycolic acid)
PTX	paclitaxel
PTX/NCTD-APRPG-NPs	PTX/NCTD-loaded core&ndash;shell lipid nanoparticles modified with APRPG
PTX/NCTD-NPs	PTX/NCTD-loaded core&ndash;shell-type nanoparticles
PTX-NPs	PTX-loaded PLGA nanoparticles
PVDF	polyvinylidene difluoride
RIPA	radioimmunoprecipitation assay
SD	standard deviation
SDS-PAGE	sodium dodecyl sulfate polyacrylamide gel electrophoresis
WB	Western blot
