# Supplementary material for: A new core–shell-type nanoparticle loaded with paclitaxel/norcantharidin and modified with APRPG enhances anti-tumor effects in hepatocellular carcinoma
Source: Front Oncol. 2022 Sep 14;12:932156. doi: 10.3389/fonc.2022.932156 (PMC9515951; doi:10.3389/fonc.2022.932156)
Supplement: Supplementary file 1 [file DataSheet_1.docx]

**Supplementary Information**

**Supplementary Materials and Methods**

***Cell Counting Kit-8 assay***

The Cell Counting Kit-8 (CCK-8, MedChemExpress LLC, Shanghai, China) was used to determine the cell viability of human hepatocyte L02 cells and hepatoma Huh-7 and Hep3B cells following the manufacturer’s instructions. In brief, CCK-8 (10 μL) was added to each well of the 96-well plates containing cultured cells, and the absorbance was recorded at 450 nm using a Thermo-max microplate reader (Thermo Fisher Scientific, Waltham, MA, USA).

***Histological examinations***

Upon completion of the experiments in the tumor-bearing mouse model established by subcutaneous injection of HepG2 cells in BALB/c nude mice, the mice were sacrificed and the tumor tissues were removed for subsequent histological examinations. In brief, paraffin-embedded tumor tissue sections were stained with hematoxylin and eosin (H&E), visualized under a microscope, and reviewed by independent pathologists. Tumor tissues were histologically evaluated and graded using the following scoring system: 0, absence of histopathological change; 1, less than 25% of histopathological changes; 2, 25–50% of histopathological changes; 3, 51–75% of histopathological changes; and 4, greater than 75% of histopathological changes.

**Supplementary Tables**

**Suppl.Table1 The particle size, drug loading, and zeta potential of PTX-NPs, PTX/NCTD-NPs, and PTX/NCTD-APRPG-NPs**

|  | Particle size | Drug loading (%) | zeta potential |
| --- | --- | --- | --- |
| PTX-NPs | ~100nm | 5.19士0.17 | 58.79－66.44mV |
| PTX/NCTD-NPs | ~110nm | 5.29士0.38^#^ | 57－60.4mV |
|  |  | 9.58±0.09^##^ |  |
| PTX/NCTD-APRPG-NPs | ~350nm | 4.95士0.13^#^ | 40.6－46.2mV |
|  |  | 10.12士0.16^##^ |  |

Note: #Loading content of PTX；##Loading content of NCTD.

**Supplementary Figures**

**Suppl. Figure 1**

**A**

**
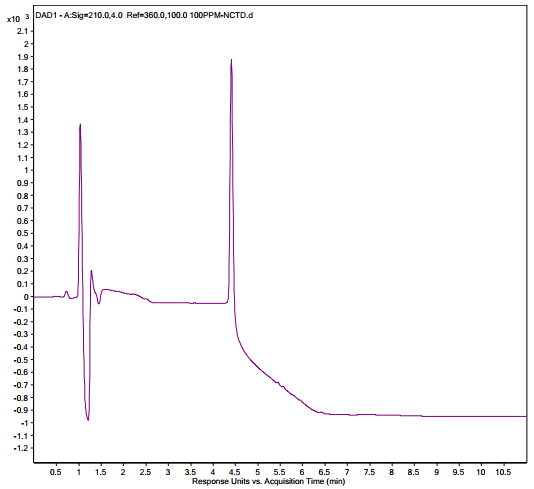
**

**B**

**
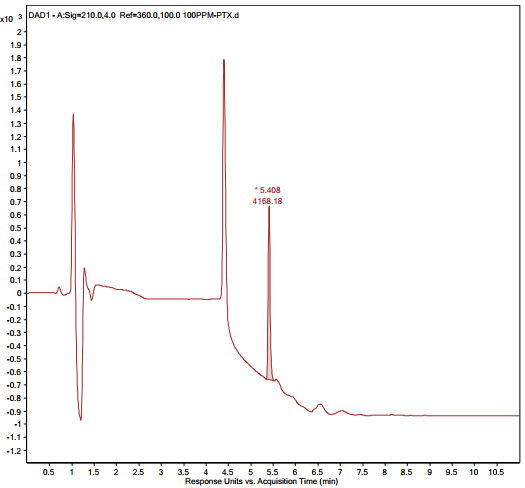
**

**C**

**
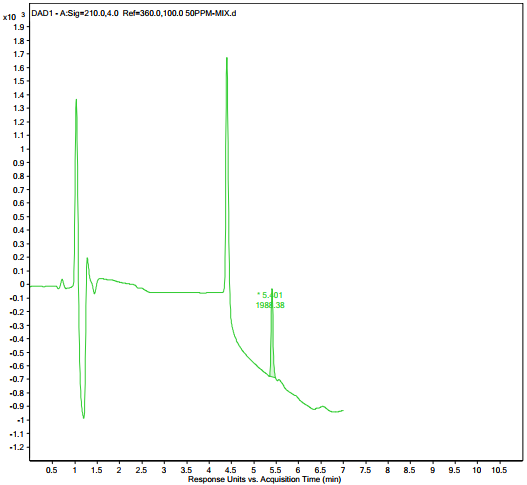
**

**D**

**
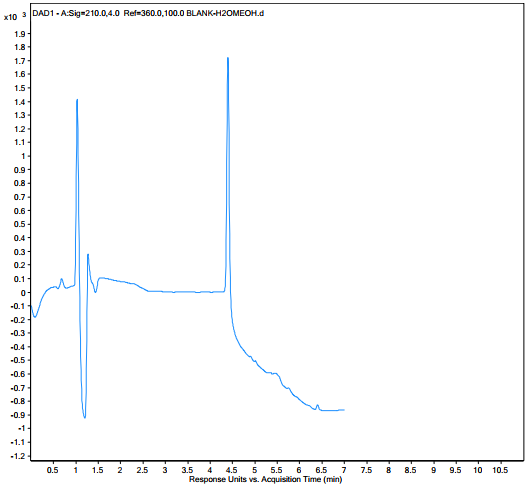
**

**Suppl. Figure 1 HPLC chromatograms.** HPLC chromatograms of (A) NCTD, (B) PTX, (C) PTX + NCTD, and (D) blank.

**Suppl. Figure 2**

**
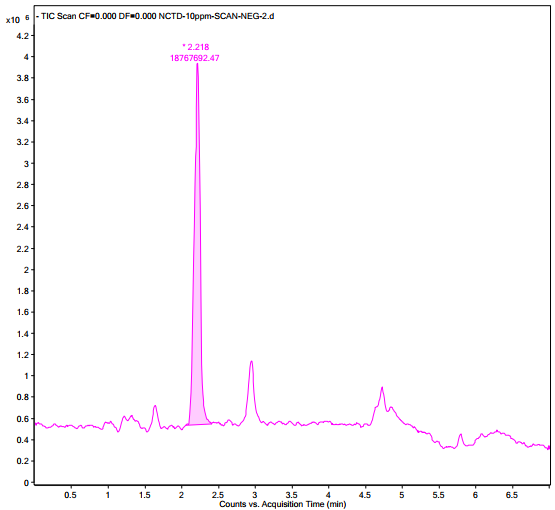
**

**Suppl. Figure 2 Total iron chromatogram (TIC) mass spectrometry of NCTD**

**Suppl. Figure 3**

**
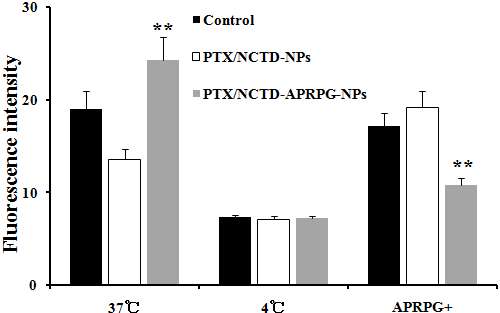
**

**Suppl. Figure 3 Quantification of fluorescence intensities of fluorescence microscopy images in HepG2 cells treated with COU6 (Control), COU6-nano (PTX/NCTD-NPs), or APRPG-COU6-nano (PTX/NCTD-APRPG-NPs) in the different groups.** HepG2 cells were incubated with water-insoluble fluorescent dye coumarin 6 (COU6, 10 mg/mL) alone as control, COU6 (10 mg/mL) in combination with PTX/NCTD-NPs (COU6-nano), or COU6 (10 mg/mL) in combination with PTX/NCTD-APRPG-NPs (APRPG-COU6-nano). After incubation for 2 h and washing three times with PBS buffer, cells were visualized under an inverted fluorescence microscope at 37ºC. The cellular uptake mechanism was investigated with a blocking experiment using free APRPG (10 mg/mL) and a low temperature test was conducted at 4ºC. The fluorescence intensity was significantly higher in the APRPG-COU6-nano group than in the COU6-nano and COU6 groups at 37ºC, whereas there were no significant differences at 4ºC. After incubation with free APRPG, the fluorescence intensity was significantly lower in the APRPG-COU6-nano group than in the COU6-nano and COU6 groups. **P<0.01, PTX/NCTD-APRPG-NPs compared with PTX/NCTD-NPs or Control.

**Suppl. Figure 4**

| 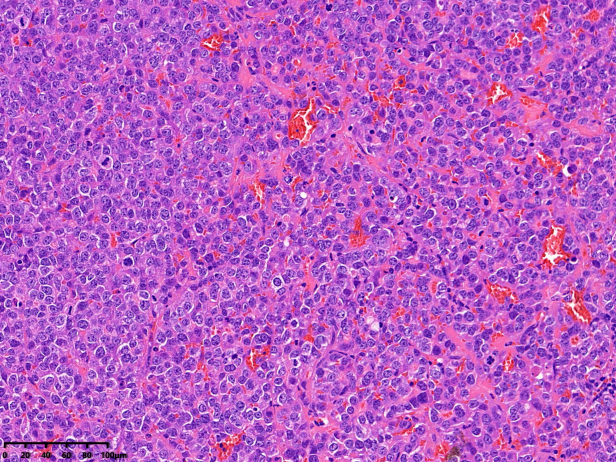 | 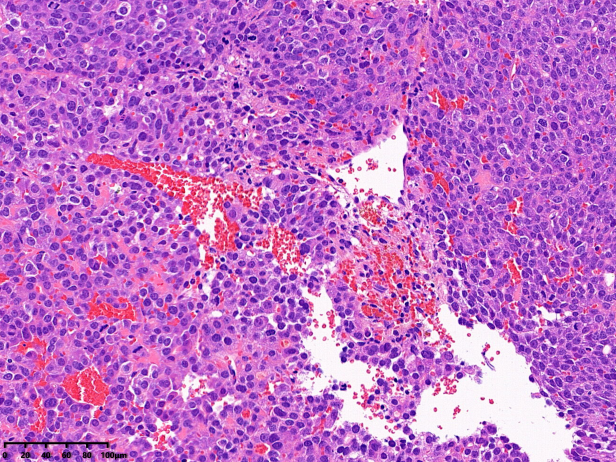 |
| --- | --- |
| Control | PTX-NPs |
| 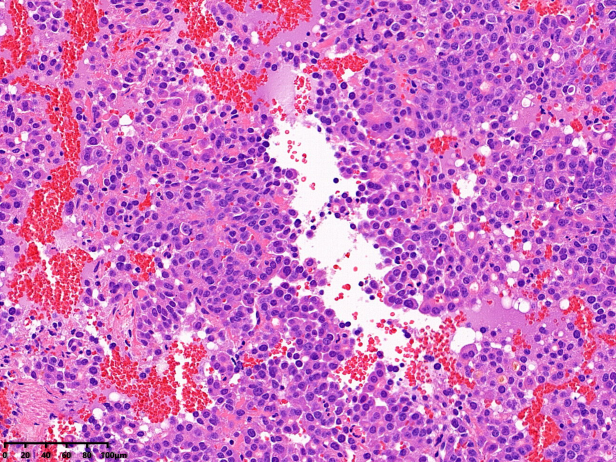 | 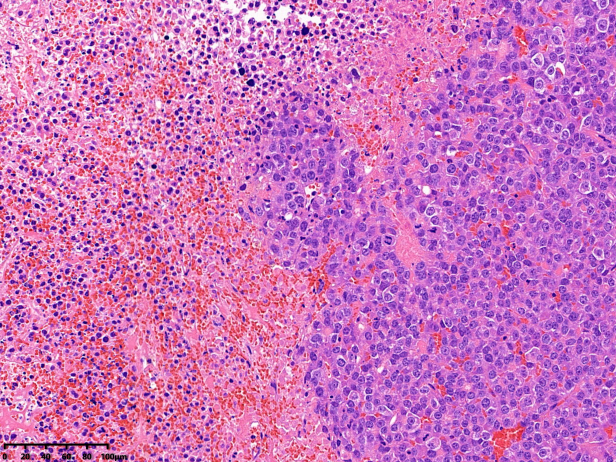 |
| PTX/NCTD-NPs | PTX/NCTD-APRPG-NPs |

**Suppl. Figure 4 Histological examinations of tumor tissues in the different treatment groups of tumor-bearing mice.** Tumor tissue sections were stained with hematoxylin and eosin (H&E) and histologically evaluated. Representative histological images of tumor tissues under 400x magnification.
